# Complete mitochondrial genome of Black Drongo *Dicrurus macrocercus* (Passeriformes: Dicruridae) and phylogenetic analyses

**DOI:** 10.1080/23802359.2021.1920490

**Published:** 2021-07-22

**Authors:** Haibo Zhang, Xue Gou, Shize Li, Cheng Wang, Caichun Peng, Canshi Hu, Mingming Zhang, Lifei Yu, Haijun Su

**Affiliations:** a College of Life Sciences, Guizhou University, Guiyang, China; b Aha Lake National Wetland Park, Guiyang, China; c Forestry College, Guizhou University, Guiyang, China; d Research Center for Biodiversity and Natural Conservation, Guizhou University, Guiyang, China; e Department of Food Science and Engineering, Moutai Institute, Renhuai, China

**Keywords:** Complete genome, gene arrangement, Black Drongo, mitochondrial DNA, *Dicrurus macrocercus*

## Abstract

The Black Drongo *Dicrurus macrocercus* is a bird belonging to the group Passeriformes, distributed almost all over the country in China. The conservation status of this species is Least Concern (LC) in IUCN. In this study, the complete mitogenome of *D. macrocercus* was determined. The mitochondrial DNA is packaged in a compact 17,056 based pair (bp) circular molecule with A + T content of 57.04%. It contains 37 typical mitochondrial genes, including 13 protein-coding genes, 2 rRNAs and 22 tRNAs, and 1 non-coding regions. We reconstructed a phylogenetic tree based on mitogenome sequences of 15 Dicruridae species and one outgroup. In trees, *D. macrocercus* was clustered as an independent clade with high support value (100). The new mitogenome data would provide useful information for application in conservation genetics and further clarify the phylogenetic evolution of this species.

The Black Drongo *Dicrurus macrocercus* is a bird with a black body and a blue-green metallic luster, which belongs to the group Passeriformes. There are seven subspecies of Black Drongo all over the world, distributed in South Asia and Southeast Asia, and three subspecies distributed in China, southern Tibet subspecies (*D. macrocercus albirictus*), Taiwan subspecies (*D. macrocercus harterti*), and common subspecies (*D. macrocercus cathoecus*). The conservation status of this species is Least Concern (LC) in IUCN. In China, the species also has been listed as a Least Concern (LC) species by the red list of China’s vertebrates (Jiang et al. 2016). This species has an extremely large range, and, hence, does not approach the thresholds for Vulnerable under the range size criterion, the population size has not been quantified, but it is not believed to approach the thresholds for Vulnerable under the population size criterion. The Black Drongo is full migrant, breeding in Afghanistan, Guam and Northern Mariana Islands, and resident in Bangladesh, Bhutan, Cambodia, China, etc. and it is distributed almost all over the country in China, they are living in different types of shrubland, grassland, savanna or artificial, terrestrial inhabitants, and does not normally occur in forest. The Black Drongo is aggressive and fierce in nature, they live in pairs or small groups, and mainly feed on insects, such as beetles, dragonflies, cicadas, and ants, and like to rest on tall trees or wires, fly down to catch the prey rapidly, and then fly to a high place directly (Zhao 2001).

Up to now, no complete mitochondrial genome data of *D. macrocercus* are available in the GenBank. In this study, we sequenced the complete mitochondrial genome of *D. macrocercus* (GenBank number: BankIt2405244 GZUNZ20201129001 MW307917) and examined its phylogenetic relationship with other Passeriformes species whose mtDNA data are available.

The tissues were collected from an accidental death of individual on 21 November 2019, collected in Longdongbao Airport of Guiyang, Guizhou Province, China, the sample was stored under the number GZUNZ20201129001 at the Research Center for Biodiversity and Nature Conservation of Guizhou University. The extraction was performed using DNA Rapid Extraction Kit (Beijing Aidlab Biotechnologies Co., Ltd, Beijing, China) according to the kit manual. The mitochondrial genomes of *D. hottentottus* (NC_043948.1) was used to design primers for polymerase chain reaction (PCR) and used as a template for gene annotation.

The complete mtDNA sequences of the Black Drongo were 17,056 bp in length, its overall base composition was: A, 32.19%; C, 28.64%; G, 14.32%, and T, 24.85%. The A + T content was 57.04%, out the range for avian mitogenomes (51.6–55.7%; Haring et al. 2001). After quality-proofing of the obtained fragments, the two mitogenomic sequences were assembled manually using DNAstar v7.1 software (Keyser 2013). First, raw mitogenomic sequences were imported into MITOS web serves (Bernt et al. 2012) to determine the approximate boundaries of genes. Exact positions of protein-coding genes (PCGs) were found by searching for ORFs (employing genetic code 9, echinoderm, and flatworm mitochondrion). All tRNAs were identified using ARWEN (Laslett and Canbck 2008), DOGMA (Wyman et al. 2004), and MITOS. The precise boundaries of rrnl and rrnS were determined via a comparison with homologs. It has a typical circular mitochondrial genome containing 13 protein-coding genes, 22 transfer RNAs, 2 ribosomal RNAs, and 1 non-coding A + T-rich region, which is usually found in birds (Boore 1999). The order and orientation are identical with the standard avian gene order (Gibb et al. 2007). Of the 13 protein-coding genes, 12 utilize the standard mitochondrial start codon ATG; however, COI use GTG as the initiation codon. TAA is the most frequent stop codon, although COII, COIII, and ND4 end with the single nucleotide T, ND1 and ND5 end with AGA, COI end with AGG, ND6 end with TAG (Table 1).

**Table 1.** Organization of the complete mitochondrial genome of Black Drongo *Dicrurus macrocercus*.

Gene	Position	Size	Spacer (+) or overlap (–)	Codon		Anti-codon	Strand
	Start–End			Start	Stop		
tRNA-Phe	1–69	69				GAA	H
12S rRNA	70–1053	984					H
tRNA-Val	1054–1123	70				TAC	H
16S rRNA	1124–2724	1601					H
tRNA-Leu	2725–2799	75				TAA	H
ND1	2809–3786	978	9	ATG	AGA		H
tRNA-Ile	3795–3867	73	8			GAT	H
tRNA-Gln	3874–3944	71	6			TTG	L
tRNA-Met	3944–4012	69	−1			CAT	H
ND2	4013–5053	1041		ATG	TAA		H
tRNA-Trp	5053–5122	70	−1			TCA	H
tRNA-Ala	5124–5192	69	1			TGC	L
tRNA-Asn	5203–5275	73	10			GTT	L
tRNA-Cys	5278–5344	67	2			GCA	L
tRNA-Tyr	5344–5414	71	−1			GTA	L
COI	5416–6966	1551	1	GTG	AGG		H
tRNA-Ser	6958–7031	74	−9			TGA	L
tRNA-Asp	7036–7104	69	4			GTC	H
COII	7113–7797	685	8	ATG	T		H
tRNA-Lys	7798–7867	70				TTT	H
ATP8	7869–8036	168	1	ATG	TAA		H
ATP6	8027–8710	684	−10	ATG	TAA		H
COIII	8717–9500	784	6	ATG	T		H
tRNA-Gly	9501–9569	69				TCC	H
ND3	9570–9920	351		ATG	TAA		H
tRNA-Arg	9922–9991	70	1			TCG	H
ND4L	9993–10,289	297	1	ATG	TAA		H
ND4	10,283–11,660	1378	−7	ATG	T		H
tRNA-His	11,661–11,730	70				GTG	H
tRNA-Ser	11,731–11,796	66				GCT	H
tRNA-Leu	11,796–11,866	71	−1			TAG	H
ND5	11,867–13,681	1815		ATG	AGA		H
Cytb	13,693–14,835	1143	11	ATG	TAA		H
tRNA-Thr	14,839–14,907	69	3			TGT	H
tRNA-Pro	14,919–14,988	70	11			TGG	L
ND6	14,999–15,517	519	10	ATG	TAG		L
tRNA-Glu	15,519–15,590	72	1			TTC	L
D-LOOP	15,591–17,056	1466					

The 12S rRNA is 984 bp, and the 16S rRNA is 1601 bp in length, which are located between tRNA-Phe(gaa) and tRNA-Leu(taa), and separated by tRNA-Val(tac). All tRNAs possess the classic clover leaf secondary structure, as observed in other bird mitogenomes (Bernt et al. 2012). Most of the mitochondrial genes are encoded on heavy strand (H-strand) except for ND6 and eight tRNA genes, which are encoded on light strand (L-strand) (Table 1).

We used MEGA7 to construct the phylogenetic tree using the maximum-likelihood method based on the mitogenome sequences of *D. macrocercus* and other 15 Dicruridae species and one outgroup by the maximum-likelihood method. In trees, *D. macrocercus* was clustered as an independent clade with high support value (100) (Figure 1).

**Figure 1.**
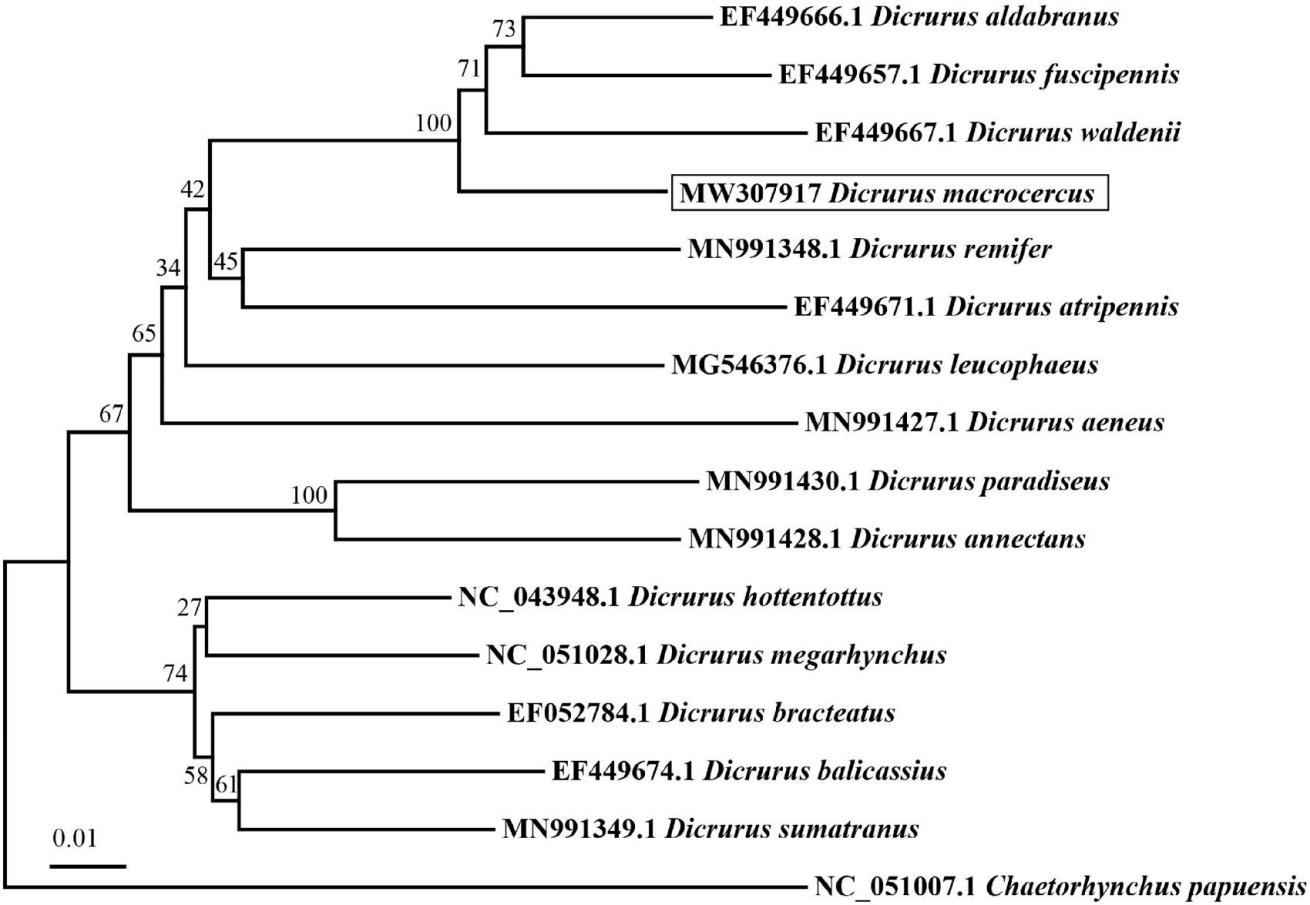
Maximum-likelihood tree based on mitogenome sequences of 15 Dicruridae species and one outgroup. Numbers nodes are bootstrap supports.

This study is the first to report and analyze the complete mitochondrial genome of Black Drongo *D. macrocercus*. The complete mitogenome of *D. macrocercus* is in favor of conservation of the non-flagship species, and provides fundamental genetic data for the evolutionary research of Passeriformes.

## Data Availability

Mitogenome data supporting this study are openly available in GenBank at https://www.ncbi.nlm.nih.gov/nuccore/MW307917; Figure, https://doi.org/10.6084/m9.figshare.13377656.
